# Feasibility of markerless real‐time tumor tracking volumetric modulated arc therapy using diaphragm‐based respiratory offset vectors

**DOI:** 10.1002/mp.70190

**Published:** 2025-12-08

**Authors:** Yukine Shimizu, Noriko Kishi, Takashi Mizowaki, Mitsuhiro Nakamura

**Affiliations:** ^1^ Department of Advanced Medical Physics Graduate School of Medicine Kyoto University Kyoto Japan; ^2^ Department of Radiation Oncology and Image‐Applied Therapy Graduate School of Medicine Kyoto University Kyoto Japan

**Keywords:** epipolar geometry, markerless real‐time tumor tracking, template matching, VMAT

## Abstract

**Background:**

Real‐time tumor tracking (RTTT) is an effective strategy for managing respiratory motion during radiation therapy; however, it typically requires invasive fiducial markers. As a non‐invasive alternative, markerless RTTT (ML‐RTTT) has gained increasing interest, especially with the growing use of volumetric modulated arc therapy (VMAT), which requires efficient real‐time motion compensation.

**Purpose:**

This study aimed to develop and evaluate a tumor position estimation algorithm for ML‐RTTT integrated with VMAT (ML‐RTTT‐VMAT), using the diaphragm dome projection as a surrogate, with an added method to compensate for phase‐dependent asynchrony between diaphragm and tumor motion.

**Methods:**

The analysis included 43 sessions performed on 23 patients whose tumors exhibited motion greater than 10 mm and who had fiducial markers implanted near the tumors. Rotational kV x‐ray images were acquired from two orthogonal directions during free breathing. Infrared reflective (IR) markers were placed on the abdomen near or above the umbilicus to track respiratory motion. Diaphragm templates were generated from planning CT images, and diaphragm positions were identified by template matching with epipolar geometry. The 3D diaphragm dome position was computed via triangulation. The initial 20 s of each session were used to build a prediction model linking the IR marker signal to tumor position. Tumor position was approximated using the centroid of the implanted fiducial markers (pseudo tumor). Three scenarios were evaluated: (i) S_no_, a baseline scenario assuming direct availability of the pseudo‐tumor position without diaphragm detection or offset‐vector estimation; (ii) S_con_, a constant offset vector defined at an end‐expiratory phase and applied to all phases; and (iii) S_var_, a variable offset vector dependent on respiratory phase. The model was validated using the remaining 50 s of data, and the prediction accuracy of each model was assessed using the 90th percentile error (*E*_90_) in each direction.

**Results:**

The *E*_90_ values for left–right, superior–inferior, and anterior–posterior directions were as follows: S_no_: 2.4, 4.1, and 4.0 mm; S_con_: 2.7, 5.7, and 4.5 mm; and S_var_: 2.3, 4.5, and 3.7 mm. Large errors were observed in six sessions (14.0% of all S_var_ sessions), mainly due to irregular respiratory patterns or pulsation artifacts in the IR marker signal.

**Conclusions:**

The proposed ML‐RTTT‐VMAT approach using diaphragm‐based prediction with a phase‐dependent offset vector is feasible and holds promise for markerless real‐time motion management in radiation therapy.

## 1 INTRODUCTION

Real‐time tumor tracking (RTTT) is an advanced technique in radiation therapy for managing respiratory motion. In RTTT, the beam's position is continuously adjusted in real time to match the tumor's movements for precise, tumor‐focused beam delivery. This method reduces the need to irradiate the entire range of tumor motion, thus minimizing the risk of adverse effects from high‐dose exposure to surrounding healthy tissues.^1^ Moreover, RTTT enhances treatment effectiveness and patient comfort by eliminating the need for breath‐hold and reducing treatment duration.^2^, ^3^, ^4^, ^5^

In RTTT radiotherapy, a hybrid approach is commonly used, wherein a correlation model, namely a four‐dimensional model (4DM), is constructed that correlates the tumor position with external respiratory signals immediately prior to beam delivery.^5^, ^6^, ^7^ The tumor position used for constructing this model is typically derived from kilovoltage (kV) x‐ray imaging. During beam delivery, the model predicts the future tumor position based on real‐time external respiratory signals, allowing the beam to be dynamically adjusted accordingly.^1^ A key advantage of this approach is that it eliminates the need for frequent continuous detection of tumor position using kV imaging during beam delivery, thereby reducing delays caused by image acquisition and processing. Furthermore, by decreasing the frequency of continuous kV imaging, the cumulative imaging dose to the patient can be reduced. While the exact dose reduction depends on the imaging protocol, previous studies have shown that continuous imaging during treatment can contribute a dose of several cGy. For instance, Nakamura et al. reported a notable reduction in imaging dose when the imaging frequency was lowered.^8^ Moreover, if 4DM is not employed, high‐frame‐rate imaging is required to determine tumor position, further increasing radiation exposure. Our method, therefore, has the potential to reduce radiation exposure associated with imaging, improving patient safety.

To develop the 4DM, accurate determination of the tumor's position from imaging is essential. However, due to variables such as tumor size, density, and overlap with the adjoining anatomical structures, direct tumor detection is challenging.^9^ To address these limitations, fiducial markers are often implanted near the tumor as detection aids. Although effective, this approach is invasive and carries risks such as marker displacement, pneumothorax, and bleeding, which can compromise treatment continuity.^10^ In response to these issues, markerless RTTT (ML‐RTTT) radiotherapy has emerged as a viable, non‐invasive alternative, attracting increasing attention within the field.^11^

Several methods for ML‐RTTT radiotherapy have been developed.^11^ Commercially available systems, such as the Xsight Lung Tracking System, integrated into the CyberKnife (Accuray, Incorporated, Sunnyvale, CA, USA),^9^ and the ExacTrac ML‐RTTT module designed for Vero4DRT (Brainlab, Incorporated, München, Germany; Hitachi High‐Tech, Tokyo, Japan)^12^, ^13^ are available. These systems, however, are suitable only for cases with high contrast between the tumor and adjacent organs. Although several artificial intelligence (AI)‐based methods for tumor detection have been proposed,^14^, ^15^ their clinical implementation faces significant legal and regulatory challenges related to AI‐driven beam delivery, particularly regarding transparency and accountability concerns.^16^, ^17^

A non‐AI approach to realizing ML‐RTTT radiotherapy is the use of the diaphragm as a surrogate for the tumor. Located at the thoracoabdominal boundary, the diaphragm demonstrates motion that is highly correlated with that of thoracoabdominal tumors,^12^ making it a promising candidate for a reliable and non‐invasive surrogate.^12^, ^18^, ^19^ However, previous studies utilizing the diaphragm have not incorporated tumor position prediction, limiting their direct applicability in clinical practice. Moreover, this approach has not been adapted for use in advanced treatment techniques such as volumetric modulated arc therapy (VMAT), in which the field of view of kV x‐ray imaging continuously rotates during beam delivery, further limiting its applicability in modern radiotherapy.

Another major challenge in estimating tumor positions from surrounding anatomical structures lies in phase‐dependent asynchrony between the tumor and surrogate, which introduces uncertainties in target localization. This phenomenon has been reported when fiducial markers are used as surrogates,^20^ and a similar issue is expected when relying on diaphragmatic motion.

In this study, we developed an algorithm to estimate tumor positions for ML‐RTTT with VMAT (ML‐RTTT‐VMAT) using the diaphragm as a surrogate marker, while explicitly accounting for the asynchrony between the tumor and diaphragm. The estimated tumor positions were then used as input to the 4DM, and the effectiveness of ML‐RTTT‐VMAT was comprehensively evaluated.

## 2 METHODS

### 2.1 Patient characteristics and data acquisition

This study was approved by the Medical Ethics Committee of the Graduate School of Medicine and Faculty of Medicine, Kyoto University, and was authorized by the head of the research institution (R1446‐2). The analysis included 43 sessions from 23 patients whose tumors exhibited motion greater than 10 mm and who had fiducial markers implanted near the tumors approximately 10 days prior to CT simulation. These patients were selected based on specific criteria and provided informed consent for data acquisition. In 19 patients with lung cancer, 2–4 spherical gold markers (1.5 mm in diameter; Olympus, Tokyo, Japan) were implanted using a bronchoscope. In 4 patients with liver cancer, a single gold coil marker (10 mm in length and 0.5 mm in diameter; IBA Dosimetry, Louvain‐la‐Neuve, Belgium) was implanted via the percutaneous approach. The inclusion criteria were as follows: availability of respiratory phase CT images, successful detection of fiducial markers and infrared reflective (IR) marker data, and consistent visibility of the diaphragm within the imaging field. All patients were treated with stereotactic body radiotherapy. Immobilization was performed using the BodyFix system (Elekta AB, Stockholm, Sweden) in the supine position with both arms raised. CT simulation was conducted using either a LightSpeed RT (GE Healthcare, Chicago, IL, USA) with a slice thickness of 2.5 mm or a SOMATOM Definition (Siemens Healthineers, Erlangen, Germany) with a slice thickness of 2 mm. Four‐dimensional CT (4DCT) was acquired using standard clinical protocols at each participating institution. Patient characteristics are summarized in Table 1. The data acquisition conditions are described in detail in reference.^21^

**TABLE 1:** Patient characteristics.

Age (median) [years]	79 (range: 65–88)
Sex (male/female)	18 / 5
Tumor stage (T1a / T1b / T2 / T2a / T4 / Meta)	9 / 7 / 2 / 2 / 1 / 2
Tumor site (left lung / right lung / left lobe of liver / right lobe of liver)	7 / 12 / 1 / 3
PTV size (median) [mL]	45.0 (range: 17.7–146.7)
Median tumor motion range [mm]	LR: 2.1 (IQR: 1.0–3.4) SI: 14.9 (IQR: 10.8–22.0) AP: 4.2 (IQR: 2.3–7.1)
Prescribed dose (40 Gy (5 fr.) / 42 Gy (14 fr.) / 48 Gy (4 fr.) / 50 Gy (4 fr.) / 60 Gy (8 fr.) / 60 Gy (16 fr.) / 70 Gy (4 fr.))	3 / 1 / 1 / 13 / 1 / 1 / 3

Abbreviations: AP, anterior–posterior; IQR, interquartile range; LR, left–right; PTV, planning target volume; and SI, superior–inferior.

For research purposes unrelated to treatment, one or two rotational kV x‐ray imaging sessions were performed on these patients from two orthogonal directions during free breathing using the Vero4DRT (Hitachi High‐Tech) under the conditions described below. The Vero4DRT has two sets of orthogonal kV x‐ray imaging systems. Imaging was performed for 70 s, with kV x‐ray frames acquired every 0.2 s. The gantry rotation angles were set to 330°–75° (clockwise) for patients with left lung cancer and 30°–285° (counterclockwise) for all other patients. The pixel size at the isocenter was 0.2 mm. Four IR markers placed on the patient's abdomen near or above the umbilicus were simultaneously tracked in the anteroposterior (AP) direction using a movable Polaris Spectra infrared camera (Northern Digital Inc., Ontario, Canada) at a sampling rate of 0.2 s. This optical tracking system, positioned close to the patient, operated independently of the Vero4DRT system. The average displacement of these four IR markers was calculated and used as the IR marker signal.

### 2.2 Determination of the pseudo‐tumor position

To establish a ground truth for subsequent analysis, the centroid of the three‐dimensional (3D) fiducial marker positions was computed and used as a proxy for the pseudo‐tumor. The two‐dimensional (2D) positions of the fiducial markers were semi‐automatically detected on all frames of the kV x‐ray image sequences. The 3D positions of the markers were then reconstructed using a triangulation method^22^ based on kV x‐ray images acquired from two orthogonal angles. The 3D position of each marker was determined as the intersection point of the rays extending from each kV x‐ray source to the corresponding 2D marker position on the imager. In cases where the back‐projected rays from the two imaging directions did not intersect, a skew configuration was formed. This situation did not arise from systematic calibration errors of the imaging system, which are typically maintained within sub‐millimeter accuracy, but rather from small uncertainties such as marker detection variability and numerical precision in reconstruction. In such cases, the midpoint of the shortest connecting segment between the two rays was adopted as the estimated 3D position.

### 2.3 Experiments

**FIGURE 1:**
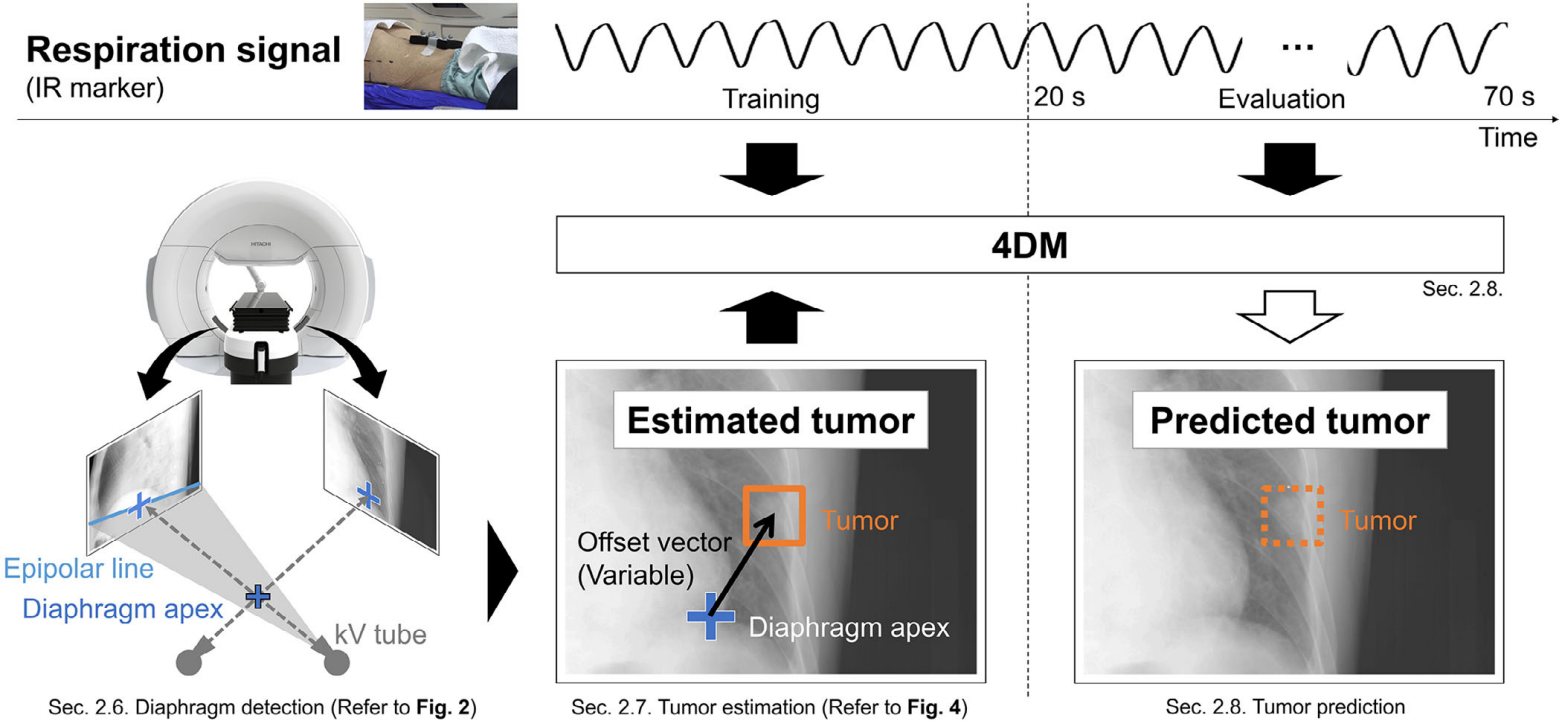
Schematic of the study process flow. The left side focuses on diaphragm detection for tumor estimation. The right side of the image shows the steps for constructing the 4DM, with black arrows indicating inputs and white arrows indicating outputs. The blue cross indicates the detection point on the diaphragm apex, and the orange rectangle represents the tumor considered in each scenario.

Figure 1 illustrates the schematic process used for the prediction of the tumor's position. This experiment was conducted on a DeepLearningBOX II/Win equipped with an Intel Core i9‐10980XE CPU (3.00 GHz), an NVIDIA RTX A5000 GPU, and 256 GB of RAM.

### 2.4 Template creation

To predict the tumor's position, the diaphragm position was first characterized using the diaphragm apex as a reference. The ipsilateral diaphragm was selected for tracking. For example, for tumors in the left lung or left lobe of the liver, the left diaphragm was tracked, and for those in the right lung or right lobe of the liver, the right diaphragm was tracked. Note that the diaphragm apex did not correspond to a unique anatomical location; rather, its projected position varied with respiratory amplitude and gantry angle. This variability could have introduced localization errors of the diaphragm apex, which in turn could have propagated into the tumor position estimation models.

Diaphragm templates were created using the planning CT (pCT), which corresponded to a specific phase of the 4DCT acquired during free breathing; the phase selected for the pCT varied among patients. To match the acquisition parameters of the kV x‐ray frames, multiple digitally reconstructed radiographs (DRRs) were generated from the same CT phase for all gantry angles at 0.3° intervals. Each DRR was cropped to a width of 299 pixels and a height of 199 pixels, with the cropping area centered at the diaphragm apex. This cropping region corresponded to a physical area of approximately 59.8 × 39.8 mm^2^ projected to the isocenter. The resulting images were stored as diaphragm templates.

### 2.5 Preprocessing before template matching

To improve diaphragm template matching, the kV x‐ray images were preprocessed to enhance contrast similarity with the DRRs. The specific preprocessing steps are described below; logarithmic transformation and density inversion were applied to adjust the overall intensity distribution and visual characteristics of the images. To eliminate the influence of fiducial markers on image processing, a simple inpainting technique was applied using the known 2D positions of the markers. Specifically, each marker region was replaced with the mean intensity value of its surrounding neighborhood. Pixel values were sampled from an annular region extending 5–10 pixels radially outward from the center of each marker. The average of these values was then used to fill the circular region within a 5‐pixel radius centered on the marker, thereby achieving smooth local intensity replacement while preserving the surrounding image structure. To enhance visual similarity to clinical x‐ray images, a logarithmic transformation was applied to compress the dynamic range, followed by density inversion to match the intensity polarity, in which projections through denser anatomical structures appeared brighter. To reduce resolution mismatch and improve the consistency of template matching between the DRRs and kV x‐ray images, the original 768 × 1024‐pixel fluoroscopic images (with a pixel spacing of approximately 0.2 mm at the isocenter) were downsampled to 75 × 205 pixels. This corresponded to a reduction by approximately a factor of 10 along the horizontal axis (orthogonal to the superior–inferior direction) and a factor of 5 along the vertical axis (aligned with the superior–inferior direction), approximating the voxel resolution of the planning 4DCT (0.98–1.17 mm and 2.0–2.5 mm, respectively). The images were then resized back to their original dimensions using bilinear interpolation to maintain compatibility with the template matching process. The processed images were then employed for diaphragm detection.

### 2.6 Diaphragm matching process

The 2D positions of the diaphragm were determined by matching preprocessed kV x‐ray images with diaphragm templates corresponding to each projection angle. The position that maximized the normalized cross‐correlation, γ(u,v), was defined as the diaphragm apex. During the training period (further details are provided in Section 2.7), visual confirmation of successful detection was performed in representative cases, after which automatic processing was applied to the entire dataset. The apex was assumed to undergo approximately rigid motion, and template matching was restricted to this region to mitigate the effects of non‐rigid deformation and projection angle‐dependent changes in appearance. Despite these assumptions, the apex region was empirically found to provide sufficient consistency for reliable detection across respiratory cycles.

**FIGURE 2:**
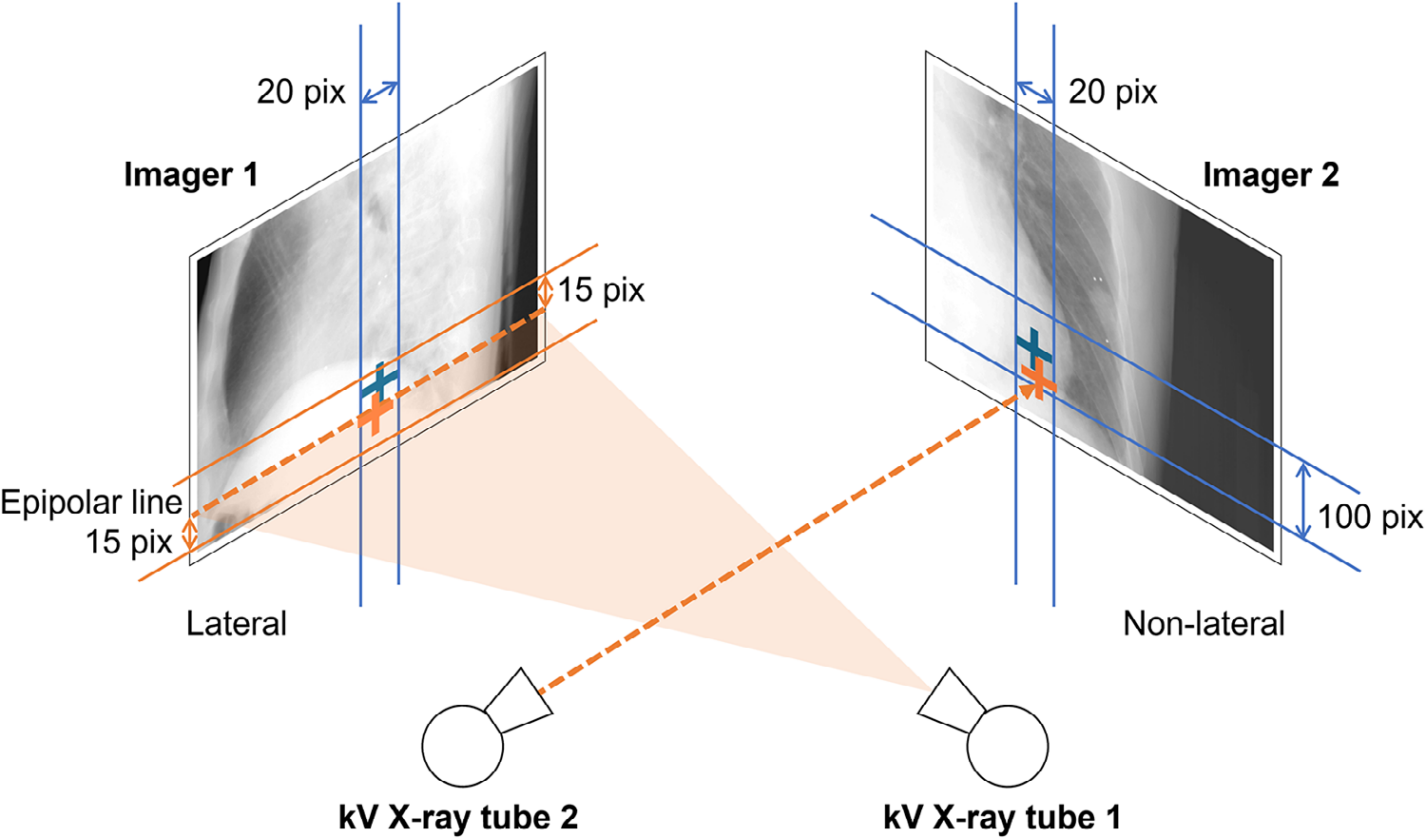
Schematic diagram of detection range determination. This diagram labels the image from Imager 1 as capturing the lateral region, while the image from Imager 2 captures the non‐lateral region. It features two pairs of kV x‐ray tubes and their corresponding imagers. Blue markers denote positions from the previous frame, and orange markers indicate positions from the current frame. Crosses indicate detection points at the diaphragm apex. Dashed lines trace the vector to the detection result in Imager 2 and its projection onto Imager 1 (the epipolar line). Solid lines outline the detection range in each direction. The scale has been adjusted for enhanced clarity.

**FIGURE 3:**
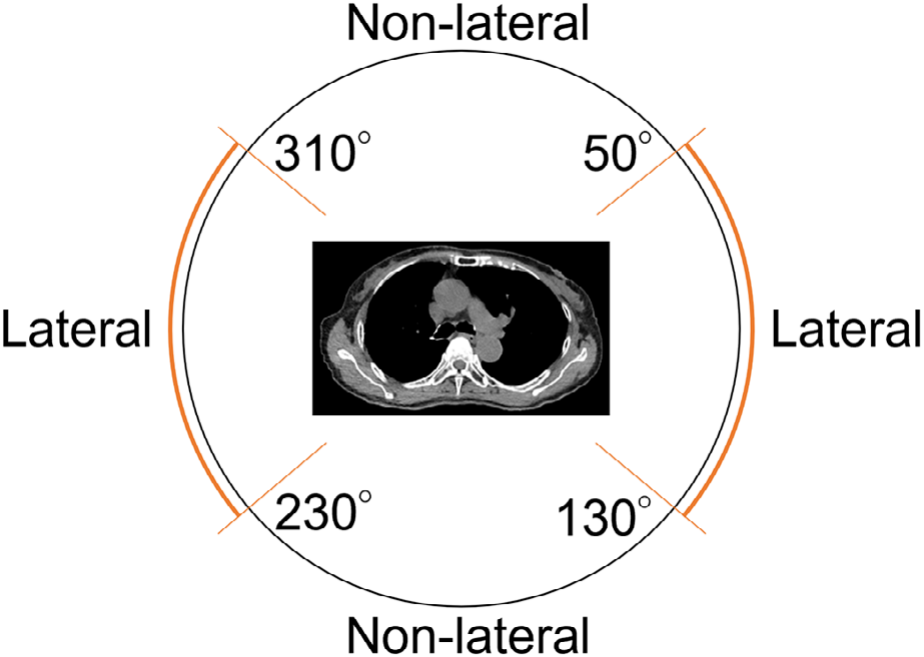
Schematic representation of the lateral and non‐lateral imaging regions. The figure shows a front view of the O‐ring gantry with the patient in the head‐first supine position. The lateral imaging region refers to the angular ranges of 50°–130° and 230°–310°, where the kV x‐ray tubes pass during gantry rotation. The remaining angles are considered the non‐lateral region.

Given the limited movement range of the diaphragm between frames, the detection range was constrained using the 2D coordinates of the diaphragm from the previous frame (*x*_pre_, *y*_pre_), as shown in Figure 2. To account for lateral displacement on the detector plane due to the rotational movement of the imaging viewpoint, the horizontal (*x*‐axis) search range—corresponding to the direction perpendicular to the superior–inferior (SI) axis—was set to 10 pixels to the left and right of *x*_pre_. The vertical (*y*‐axis) search range—aligned with the SI direction—was defined based on two categories of imaging angles. The “lateral imaging region,” encompassing angle ranges of 50°–130° and 230°–310° as shown in Figure 3, was characterized by the potential overlap of the left and right diaphragms. The other imaging angles were categorized as the “non‐lateral imaging region.” When the left or right hemidiaphragm was within the field of view in the lateral x‐ray projection (lateral imaging region), the contralateral hemidiaphragm was outside this view, within the field of the orthogonal (non‐lateral imaging region) projection.

#### 2.6.1 Non‐lateral imaging region

The diaphragm displacement between frames in the kV x‐ray images was typically less than 50 pixels, corresponding to approximately 10 mm in physical distance. Therefore, the search range was defined as 50 pixels above and below *y*_pre_.

#### 2.6.2 Lateral imaging region

In the lateral imaging region, potentially false detection of the diaphragm on the opposite side needs to be addressed. To manage this, we introduced epipolar lines—geometric constraints representing the projection of detected 3D points from the non‐lateral view onto the lateral image plane.^23^ These lines, derived from the known projection geometry and camera parameters, indicate where the corresponding structure had to lie in the lateral view. In this study, epipolar lines were used to determine the expected vertical position of the diaphragm region, and the template matching search range was restricted to within 15 pixels of the points where the lines intersected the left and right edges of the image. This constraint enhanced both detection accuracy and computational efficiency.

### 2.7 Estimation of the tumor position

In this study, the diaphragm served as a surrogate for fiducial markers to facilitate ML‐RTTT‐VMAT. However, the tumor and diaphragm often moved asynchronously across different respiratory phases.^20^ To quantify this discrepancy, we defined an offset vector as the positional difference between the diaphragm surrogate and the tumor position. Based on this definition, we established three scenarios with distinct types of offset vectors for comparison against a variable offset vector. For each session, the first 20 s were used to train the 4DM, followed by 50 s for evaluation. The 20‐second training period was selected to assess whether model construction would be feasible within a clinically practical timeframe. The offset vector was determined during the training period. The details are provided below.

#### 2.7.1 Scenario S_no_: No use of offset vectors (only use pseudo tumor)

To evaluate the intrinsic prediction error of the 4DM model, a baseline scenario was established in which the pseudo‐tumor position was directly provided to the 4DM without diaphragm detection or tumor estimation using the offset vector. This scenario assumed that the tumor position could be directly observed. However, such direct tumor identification is rarely feasible in clinical practice^9^; therefore, this baseline did not represent a realistic tracking scenario but instead served as a reference for the minimal prediction error achievable by the model. Further details of the 4DM are provided in Section 2.8.

#### 2.7.2 Scenario S_con_: Constant offset vectors

In this scenario, the offset vector, similar to that being used in clinical practice, was adopted.^24^ A constant vector from the diaphragm apex to the tumor position was defined based on the end‐expiratory phase in the training period, as identified using the IR marker signal. This vector, assumed to remain unchanged during the training period, was applied to the tracked diaphragm positions to estimate tumor location. Although this approach did not account for non‐superior–inferior motion or respiratory hysteresis, it was conceptually analogous to clinical scenarios in which tumor position is estimated from a fixed spatial relationship with a fiducial marker.

#### 2.7.3 Scenario S_var_: Variable offset vectors

This scenario represents our proposed approach. During the training period, representative end‐expiratory and end‐inspiratory phases were defined using the final trough and peak of the 20‐second respiratory waveform derived from the IR marker signal. The corresponding 3D positions of the diaphragm and tumor at these time points were extracted and used to calculate directional vectors. The intermediate respiratory phases between end‐expiration and end‐inspiration were estimated using Equation (1), which mapped the IR marker signal to a continuous respiratory phase. In clinical practice, this step is assumed to be performed by acquiring cone‐beam CT (CBCT) images under breath‐hold conditions. Although acquiring both end‐inspiratory and end‐expiratory breath‐hold CBCT scans could potentially increase patient burden, the system used in this study employed dual x‐ray tubes, enabling simultaneous acquisition and allowing completion of the CBCT scans within 16 s. This capability helped minimize patient burden during the imaging process.

A vector in the SI direction was defined at each respiratory phase, with the apex of the diaphragm serving as the starting point and the tumor position as the endpoint. This vector represented the relative displacement between the diaphragm and the tumor due to respiration. In the left–right (LR) and AP directions, the diaphragm apex during the end‐expiratory phase was used as the fixed starting point to avoid errors caused by respiratory‐induced shifts of the diaphragm in those directions. Tumor positions at each respiratory phase were used as endpoints to represent displacement relative to this stable reference, thereby minimizing inconsistencies due to lateral diaphragm motion. The relationships between diaphragm and tumor positions were modeled separately in each direction (LR, SI, and AP) by fitting a simple linear regression, where tumor position was expressed as a linear function of the diaphragm apex position. This empirical model, represented by the following equation, captured the relative displacement between the diaphragm and tumor during respiration:

(1) $$\begin{array}{c} s=\frac{s_{\mathrm{ex}}-s_{\mathrm{in}}}{p_{ex}-p_{in}}\;\cdot p+\left(s_{\mathrm{in}}-\frac{s_{\mathrm{ex}}-s_{\mathrm{in}}}{p_{ex}-p_{in}}\cdot p_{in}\right), \end{array}$$

where ***s*** represents the offset vector for each respiratory phase, and *p* represents the AP centroid of the IR marker signal, defined as the average position of four IR markers projected onto the AP axis at each time point. The subscript “in” denotes the end‐inspiration, and “ex” denotes the end‐expiration.

**FIGURE 4:**
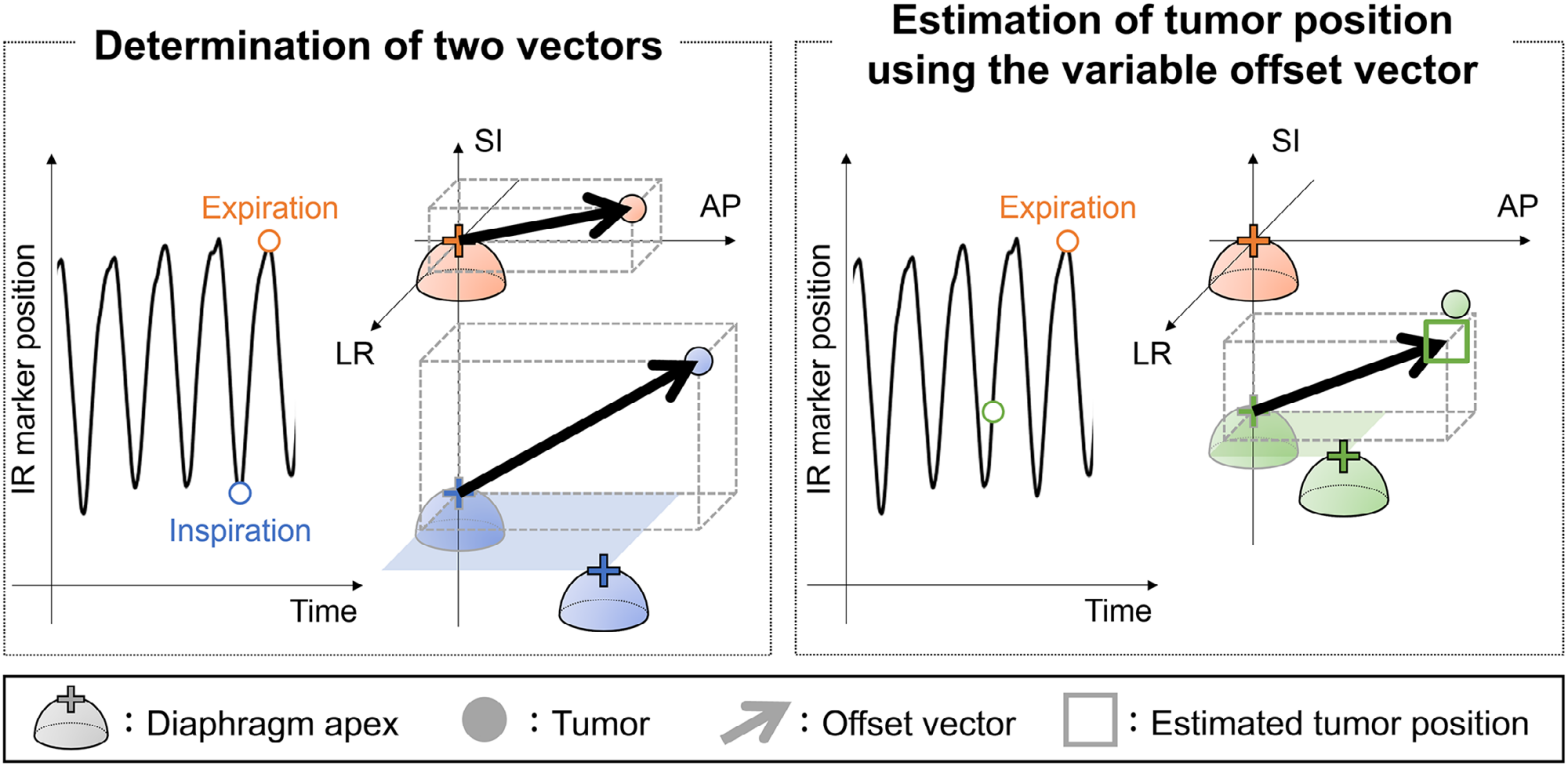
Schematic diagram illustrates the determination of the variable offset vector and tumor position estimation. The coordinate system of the IR marker position is defined that the positive IR marker position corresponds to posterior displacement relative to the anterior.

During the training period, these functions were then applied to diaphragm detection coordinates across all frames to estimate tumor positions, including under irregular breathing conditions. The starting point for each directional calculation was consistent with the function creation process. Figure 4 demonstrates the process for determining the variable offset vector and the tumor position based on this vector.

### 2.8 Prediction of tumor position

To predict tumor position during the evaluation period, the 4DM was constructed during the training period using regression between the IR marker signal and tumor position, as defined in the scenario,^5^, ^25^ and is mathematically expressed as a quadratic function, as follows:

(2) $$\begin{array}{c} F\left(p\right)=ap^{2}+bp+c+d{\dot{p}}^{2}+e\dot{p}, \end{array}$$

where *p* represents the centroid position in the AP direction of the IR marker signal, $\dot{p}$ represents the velocity in the AP direction of the IR marker signal, and *a*, *b*, *c*, *d*, and *e* are the model parameters optimized for each IR marker and tumor position using a least squares gradient‐based method. During the prediction phase of the testing period for S_no_, S_con_, and S_var_, the predicted tumor positions were calculated by inputting the average positions and velocities of the IR markers into the model.

### 2.9 Evaluation

To assess the necessity of incorporating the respiratory offset vector approach, alignment accuracy was compared between conditions with and without offset correction. Specifically, the 3D positions of the diaphragm apex in the end‐inspiratory phase were first shifted to align with those in the end‐expiratory phase. To assess the differential motion between the pseudo‐tumor and diaphragm across respiratory phases, the vector displacement of the diaphragm apex between the end‐inspiratory and end‐expiratory phases was subtracted from the tumor motion. The resulting residual vector represents the tumor motion relative to the diaphragm. This step was not part of the model‐building process but was performed to illustrate the rationale for applying the offset vector approach.

Next, the prediction errors in each scenario were calculated using the pseudo‐tumor (the centroid of the gold markers) as the ground truth. For the LR, SI, and AP directions, cumulative frequency distributions of positional errors were generated using data from all time points across all sessions. The 90th and 95th percentile errors (*E*_90_ and *E*_95_, respectively) values were then extracted from each distribution as indicators of the upper error bound. To evaluate the significance of the differences among the *E*_90_ values of the different scenarios (S_no_, S_con_, and S_var_) and directions (LR, SI, and AP), a two‐way analysis of variance (ANOVA) was performed for all sessions, with the significance level set at 0.05.

Subsequently, in addition to the overall error evaluation, session‐specific performance was assessed by calculating the *E*_90_ values for each session. To evaluate tracking efficiency for S_var_, the reduction ratio (*R*) was defined for each session as follows:

(3) $$\begin{array}{c} R=1-\frac{E}{A}, \end{array}$$

where *E* represents the *E*_90_ value in the SI direction for each session, and *A* denotes the corresponding SI displacement of the pseudo‐tumor. The median and interquartile range (IQR) of *R* were then computed.

Additionally, the motion of the IR markers was compared between the training and evaluation periods in cases where the *E*_90_ values for S_var_ exceeded the overall *E*_90_ value across all sessions. For each session, average respiratory amplitude and cycle were calculated separately for the training and evaluation periods. The average cycle was defined as the duration of each period divided by the number of inspiratory peaks. To examine the temporal consistency of the IR marker–pseudo‐tumor relationship, correlation coefficients between the IR marker and pseudo‐tumor positions were calculated for each directional component in both the training and evaluation periods. These values were used as a supplementary indicator to assess whether the surrogate–tumor relationship changed substantially over time, rather than as a direct measure of model accuracy.

## 3 RESULTS

### 3.1 Phase‐dependent asynchrony between the tumor position and the diaphragm apex

The comparison of 3D tumor positions across respiratory phases revealed a median residual displacement of 6.1 mm (IQR: 3.8–8.5 mm) after the diaphragm apex positions in the end‐inspiratory phase were shifted to align with those in the end‐expiratory phase. This residual distance represents the discrepancy between tumor and diaphragm motion, independent of diaphragm alignment.

Among sessions involving lung cancer patients (36 sessions), the median 3D distance was 5.8 mm (IQR: 3.7–7.5 mm). In contrast, sessions involving liver cancer patients (seven sessions) showed a median 3D distance of 8.3 mm (IQR: 6.4–10.3 mm). These findings highlighted the necessity of the variable offset vector.

### 3.2 Overall comparison

Table 2 summarizes the mean ± standard deviation (SD), and *E*_90_ and *E*_95_ values of tumor position prediction in each direction for all three scenarios (S_no_, S_con_, and S_var_). Regardless of the metric used, prediction errors were largest in the SI direction, followed by the AP and LR directions. The two‐way ANOVA results for the *E*_90_ values indicated no significant differences among S_no_, S_con_, and S_var_ (*p* = 0.09), whereas significant differences were found in the *E*_95_ values (*p* < 0.05).

**TABLE 2:** Mean ± SD, *E*_90_ and *E*_95_ values in S_no_, S_con_, S_var_. The mean ± SD values were calculated while retaining the sign of the errors, whereas *E*_90_ and *E*_95_ were derived from the absolute values.

		S_no_	S_con_	S_var_
LR	Mean ± SD [mm]	0.3 ± 1.6	−0.1 ± 2.1	−0.1 ± 1.4
	*E*_90_ [mm]	2.4	2.7	2.3
	*E*_95_ [mm]	2.9	3.7	3.0
SI	Mean ± SD [mm]	−0.6 ± 4.1	−0.3 ± 4.2	−0.7 ± 4.0
	*E*_90_ [mm]	4.1	5.7	4.5
	*E*_95_ [mm]	6.9	8.5	7.9
AP	Mean ± SD [mm]	1.2 ± 2.5	−0.3 ± 3.3	0.8 ± 2.2
	*E*_90_ [mm]	4.0	4.5	3.7
	*E*_95_ [mm]	4.6	5.6	4.5
3D	Mean ± SD [mm]	3.6 ± 3.7	4.2 ± 3.9	3.5 ± 3.4
	*E*_90_ [mm]	5.8	8.0	6.0
	*E*_95_ [mm]	8.0	11.7	8.6

A comparison of *E*_90_ and *E*_95_ across all time points showed minimal differences between S_no_ and S_var_, indicating that prediction errors associated with the variable offset vector were negligible in the LR and AP directions. In contrast, a difference in *E*_90_ and *E*_95_ was observed between S_con_ and S_var_ in the SI direction, suggesting that expansion and contraction of the offset improved prediction accuracy. By disease site, as shown in Table 3, lung tumors exhibited larger prediction errors compared with liver tumors.

**TABLE 3:** Mean ± SD, *E*_90_ and *E*_95_ values in S_no_, S_con_, and S_var_ by disease site. The mean ± SD values were calculated while retaining the sign of the errors, whereas *E*_90_ and *E*_95_ were derived from the absolute values.

		Lung (36 sessions)			Liver (7 sessions)		
		S_no_	S_con_	S_var_	S_no_	S_con_	S_var_
LR	Mean ± SD [mm]	0.2 ± 1.6	−0.1 ± 2.2	0.0 ± 1.4	0.9 ± 0.9	0.2 ± 1.5	−0.2 ± 1.3
	*E*_90_ [mm]	2.5	2.8	2.4	2.0	2.4	2.2
	*E*_95_ [mm]	3.1	4.1	3.0	2.4	2.8	2.8
SI	Mean ± SD [mm]	−0.5 ± 4.4	−0.4 ± 4.3	−0.7 ± 4.3	−0.8 ± 1.1	−0.1 ± 3.3	−1.0 ± 1.5
	*E*_90_ [mm]	4.7	5.8	5.1	2.3	5.1	2.9
	*E*_95_ [mm]	7.9	8.8	8.8	2.9	7.4	3.5
AP	Mean ± SD [mm]	0.9 ± 2.5	−0.3 ± 3.4	0.5 ± 2.2	2.8 ± 1.6	0.1 ± 3.1	2.3 ± 1.9
	*E*_90_ [mm]	3.7	4.4	3.4	4.8	4.8	4.7
	*E*_95_ [mm]	4.3	5.7	4.2	5.2	5.6	5.1
3D	Mean ± SD [mm]	3.7 ± 4.0	4.2 ± 4.2	3.6 ± 3.6	3.5 ± 1.4	4.1 ± 2.4	3.3 ± 16
	*E*_90_ [mm]	6.1	8.2	6.4	5.4	7.3	5.4
	*E*_95_ [mm]	8.9	12.6	9.6	5.8	8.9	6.0

The computation time for tumor position estimation in this scenario was approximately 100 ms per frame.

### 3.3 Session‐specific comparison

**FIGURE 5:**
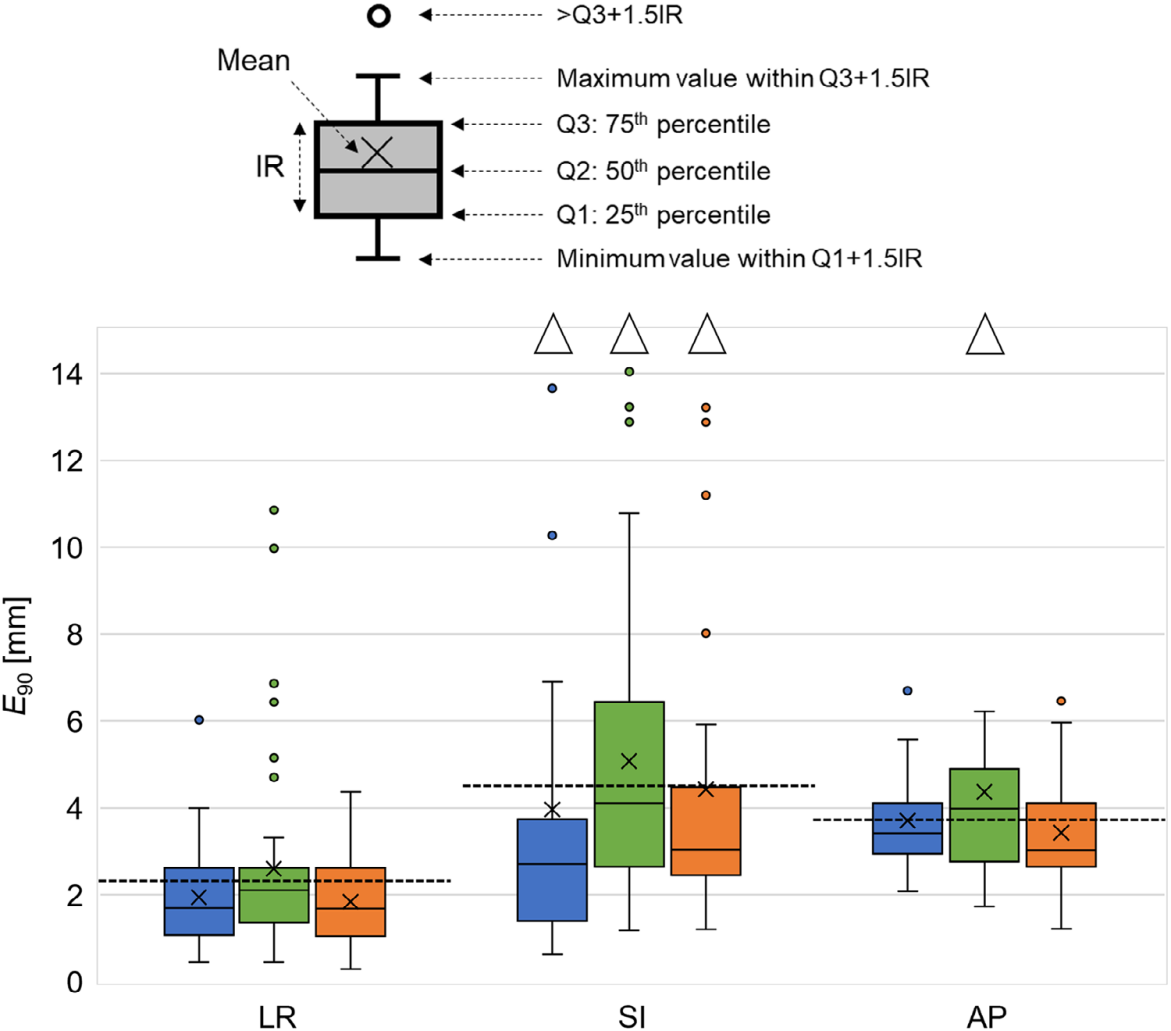
Box plots for each session. Colors blue, green, and orange denote S_no_, S_con_, and S_var_, respectively. Dashed lines represent the *E*_90_ values for all sessions in each direction in S_var_.

*E*_90_ values were calculated for each session in the LR, SI, and AP directions, and the results are presented as box plots in Figure 5. The dashed lines indicate the overall *E*_90_ values calculated across all sessions. The variation in the SI direction was more pronounced than in the LR and AP directions. In the S_var_ scenario, the *E*_90_ values in the SI direction exceeded the overall *E*_90_ value in six sessions. Notably, the overall *E*_90_ value in the S_var_ scenario appeared within the IQR of session‐wise *E*_90_ values, reflecting a skewed distribution of errors across sessions, a theoretically valid result due to differing statistical characteristics between pooled and individual data.

The median value of *R* was 81% (IQR: 72%–86%). This reflects the extent to which the irradiation margin could be reduced compared with covering the full range of tumor motion.

**FIGURE 6:**
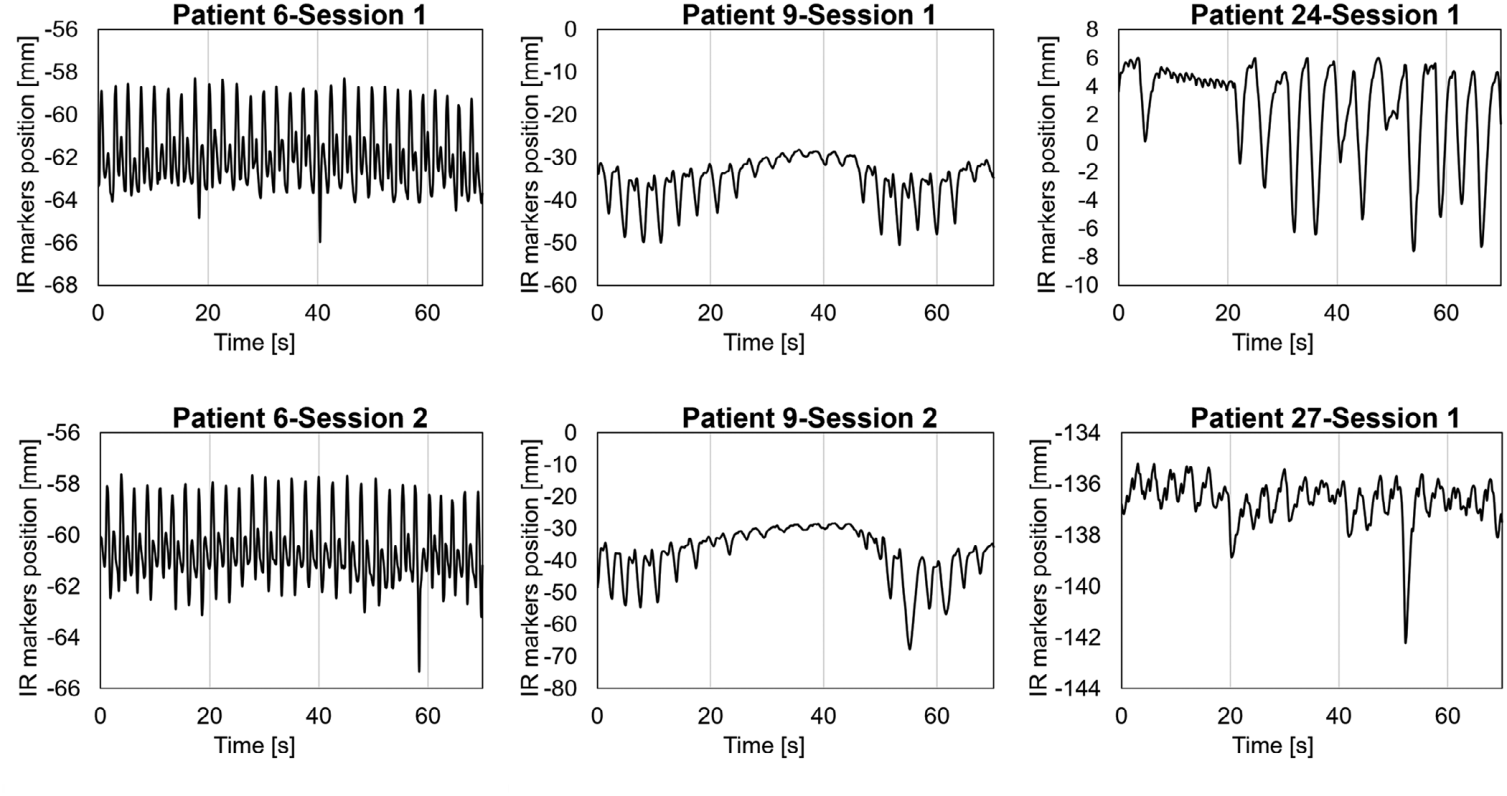
IR marker signal positions derived from actual patient respiratory traces during sessions were identified as outliers.

**FIGURE 7:**
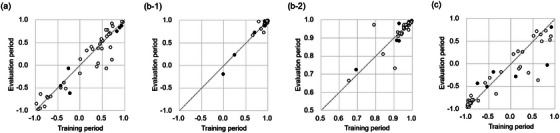
Scatter plots showing the correlation coefficients between the IR marker and tumor positions during the training and evaluation periods for each directional component: (a) left–right, (b‐1) superior–inferior, (b‐2) enlarged superior–inferior and (c) anterior–posterior. The sessions identified as outliers are marked with black circles, while the other sessions are marked with white circles. The dashed line represents the line of equality.

**FIGURE 8:**
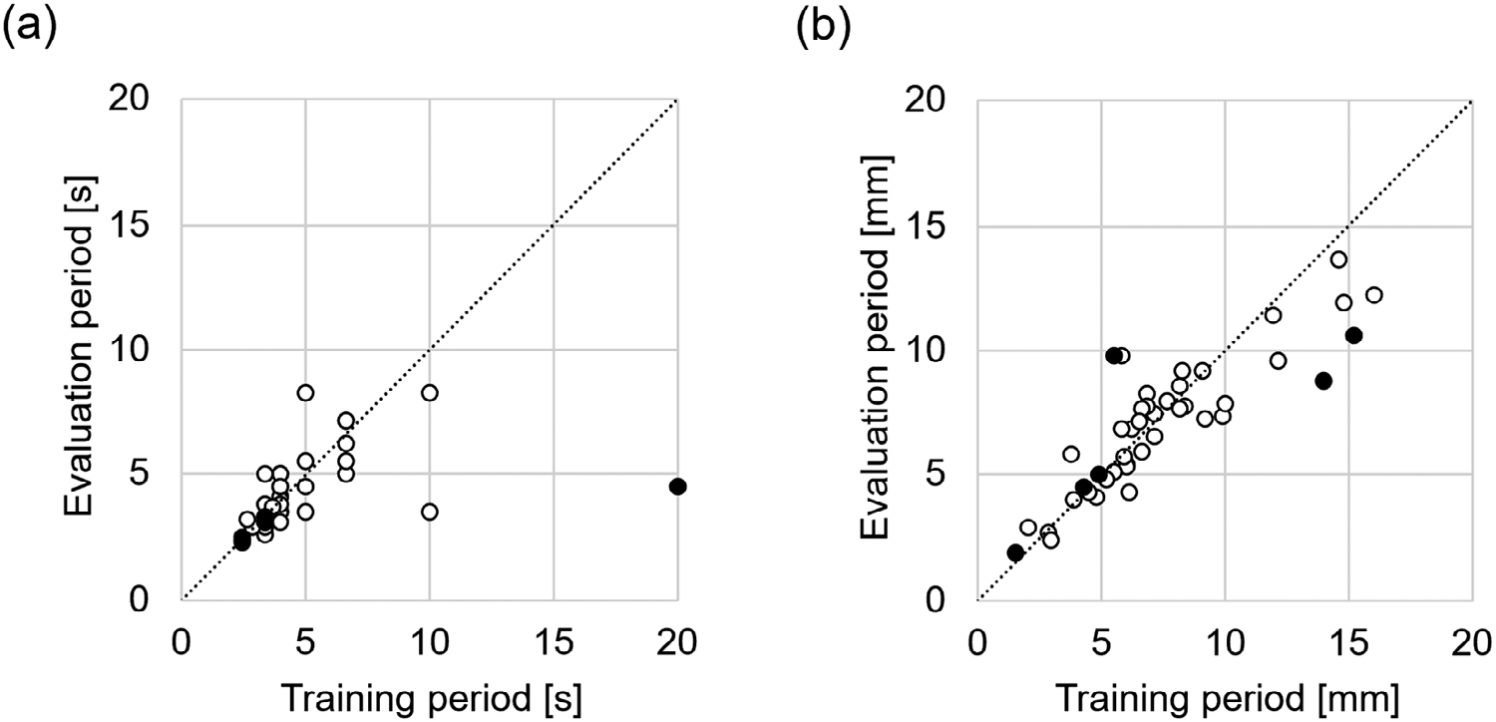
Scatter plot depicting differences in average IR marker amplitude between the training and evaluation periods. The left diagram shows the average respiratory cycle (a), and the right diagram shows the amplitude (b). The sessions identified as outliers are marked with black circles, while the other sessions are marked with white circles. The dashed line represents the line of equality.

These sessions were identified as outliers, and their *E*_90_ values are listed in Table 4. The errors in the SI direction were substantial for all sessions. The corresponding waveforms of the IR marker signals are depicted in Figure 6, revealing irregular patterns caused by pulsations or their strong influences. Figure 7 illustrates the correlation coefficients (CC) between the IR marker and pseudo‐tumor positions during the training and evaluation periods for each directional component. The scatter plots show that the CC values remained relatively consistent between the two periods across all directions. Although CC does not fully represent model accuracy, this result indicates that the surrogate–tumor relationship did not exhibit marked temporal variation, thereby supporting the validity of using the training data to construct the motion model. Figure 8 presents a scatter plot that compares the average amplitude and cycle of IR marker motion between the training and evaluation periods. Notable deviations in the average respiratory cycles were observed among the outlier sessions. Only a small fraction of these outliers exhibited differences in cycle or amplitude between the training and evaluation periods, indicating that most of the substantial errors were independent of the training versus evaluation periods.

**TABLE 4:** *E*_90_ values in S_no_, S_con_, and S_var_, and during sessions identified as outliers in the SI direction. Values exceeding the overall *E*_90_ value across all sessions are highlighted in bold. Correlation coefficients represent the relationship between the IR marker and pseudo‐tumor motion during the evaluation period.

				Correlation coefficient			S_no_ [mm]			S_con_ [mm]			S_var_ [mm]		
Patient	Session	Tumor site	PTV size [mL]	LR	SI	AP	LR	SI	AP	LR	SI	AP	LR	SI	AP
6	1	Left lung	69.3	−0.1	−0.2	−0.2	2.5	**13.7**	**5.5**	1.9	**17.1**	4.5	1.9	**13.2**	**4.5**
6	2	Left lung	69.3	−0.5	0.2	−0.5	2.4	**13.8**	**5.5**	2.1	**12.9**	5.3	2.6	**12.9**	**6.0**
9	1	Left lung	37.4	0.9	0.9	0.8	2.9	**6.9**	3.2	**6.4**	**8.7**	4.0	**3.4**	**8.0**	3.0
9	2	Left lung	37.4	0.8	0.9	0.0	**4.0**	**14.7**	2.9	**6.9**	**13.2**	2.5	**3.8**	**15.3**	2.9
24	1	Left lung	62.8	−0.6	1.0	−0.3	**6.0**	**23.9**	4.1	**5.2**	**14.0**	5.3	**4.0**	**20.6**	**5.8**
27	1	Right lung	146.7	0.8	0.7	−0.4	1.3	**10.3**	3.9	1.6	**10.8**	3.2	1.7	**11.2**	4.1
All	All	—	—	—	—	—	2.9	6.9	4.6	3.7	8.5	5.6	3.0	7.9	4.5

Abbreviations: AP, anterior–posterior; LR, left–right; PTV, planning target volume; and SI, superior–inferior.

## 4 DISCUSSION

In this study, we developed a tumor position estimation algorithm that addresses tumor–diaphragm asynchrony—defined as the phase‐dependent differences in motion between the tumor and the diaphragm—by utilizing the diaphragm as a surrogate for the tumor. This study aimed to develop a hybrid ML‐RTTT‐VMAT. The diaphragm was utilized as a surrogate for the tumor, and a position estimation method that accounts for the fact that the tumor and diaphragm may not move synchronously across different respiratory phases was proposed. In S_var_, the *E*_90_ values in the LR, SI, and AP directions were 2.3, 4.5, and 3.7 mm, respectively. When comparing *E*_90_ values across all time points, the difference between S_no_ and S_var_ was 1.0 mm in the SI direction, suggesting that the variable offset vector's impact on prediction errors was negligible. Furthermore, while the errors in S_no_ were comparable to those in S_var_, it should be noted that S_no_ assumes direct identification of the tumor position, which is rarely achievable in practice.^9^ In contrast, when the diaphragm was employed as a surrogate, errors in S_var_ were consistently smaller than those in S_con_, highlighting the effectiveness of the variable offset vector approach in clinically relevant scenarios.

Although this study validated the proposed method using fiducial markers as pseudo‐tumors, the ultimate goal is clinical implementation in a fully markerless setting. In such a scenario, tumor and diaphragm contours can be delineated from end‐inspiratory and end‐expiratory CBCT images and projected onto imaging planes to define their relative offset vectors automatically. Although not investigated in this study, 4D‐CBCT may also serve as a viable alternative for deriving these vectors. During treatment, a patient‐specific 4D model, constructed using IR marker signals and tumor motion information, enables real‐time tumor position estimation by combining the respiratory signal with predefined offset vectors. This model can incorporate short‐term prediction (e.g., 25 ms) to compensate for system latency. A brief modeling period, typically around 20 s, following CBCT acquisition, may suffice for model initialization. The model can then be applied throughout the treatment session, with periodic imaging for verification. Predefined criteria would determine when model adjustment or retraining is required, especially under conditions such as irregular breathing or baseline drift. Future studies are warranted to develop these operational protocols and assess their clinical feasibility.

This study evaluated the prediction errors of S_var_ using *E*_90_ and *E*_95_ values at all time points. Specifically, for lung tumors, *E*_90_ values in the LR, SI, and AP directions were 2.4, 5.1, and 3.4 mm, respectively, whereas for liver tumors, the corresponding errors were 2.2, 2.9, and 4.7 mm. Kito et al. previously proposed margins for lungs, liver, and pancreas in multi‐institutional phase II studies on fiducial marker–based fixed‐gantry RTTT radiotherapy.^26^ For the lungs, they suggested up to 3.2, 6.2, and 4.2 mm in the LR, SI, and AP directions, respectively, while for the liver, the recommended values were 2.1, 4.6, and 3.1 mm. Although the *E*_90_ values are not formally defined as clinical margins, their magnitudes were comparable to those reported as margins by Kito et al., suggesting that the prediction errors observed in this study could reasonably be interpreted as being within the range of clinically acceptable margins.

Sessions in which the *E*_90_ values in S_var_ exceeded the overall *E*_90_ value calculated across all time points were analyzed to identify the underlying causes. The IR marker signals in these sessions exhibited irregularities, including temporary breathing pauses, deep inspiration, amplitude changes, and baseline drift, as shown in Figure 6, which were considered contributing factors to the decline in prediction accuracy. Additionally, sessions with substantial discrepancies in IR marker signal behavior between the training and evaluation periods were identified (Figure 7). These findings underscore the strong dependence of tumor position estimation on the phase correspondence between the IR marker signal and tumor motion. Consequently, incorporating respiratory control may enhance the efficacy and precision of RTTT radiotherapy. Although these irregularities were identified retrospectively, they may also be detected in advance by analyzing the variability of the IR marker signals during the initial training period. For instance, large‐amplitude fluctuations or unstable baselines in the early waveform could indicate potential challenges in model accuracy. In clinical practice, such patterns could serve as indicators for adapting the tracking strategy, such as extending the training period, employing respiratory coaching or control, or implementing periodic imaging verification during beam delivery. In cases where deviations exceed predefined thresholds, model retraining or switching to alternative motion management techniques may be warranted. Future work is required to establish robust criteria for these adaptive interventions and to evaluate their impact on treatment accuracy.

To contextualize our findings, we calculated the mean ± SD of the tumor position prediction errors in each directional component. In our best‐performing scenario (S_var_), the errors were −0.1 ± 1.4 mm in the LR direction, −0.7 ± 4.0 mm in the SI direction, and 0.8 ± 2.2 mm in the AP direction. Although the AAPM the MArkerless Lung Target Tracking CHallenge (MATCH) study did not provide direction‐specific mean ± SD in tabulated form, overall error distributions for participating methods were reported to be within a similar range.^27^ The slightly larger errors observed in our study may reflect the broader diversity of respiratory patterns included in our dataset, such as baseline drift, amplitude variability, and pulsation‐related artifacts, conditions less represented in the MATCH study. Despite these challenges, our results support the feasibility and robustness of the proposed diaphragm‐based tracking approach in more variable and clinically realistic settings.

Table 5 provides a comparison of previous studies that have investigated tumor position errors in ML‐RTTT methods employing the diaphragm. These earlier studies utilized fixed imaging viewpoints, whereas our study adopted a technique compatible with kV x‐ray images from a rotational perspective. Rostamzadeh et al. used the ExacTrac module to predict the diaphragm position from two directions and estimated its contribution to the planning target margin using prediction errors based on the van Herk formula,^28^, ^29^ reporting margins of 2.2, 5.0, and 4.7 mm in the LR, SI, and AP directions, respectively.^12^ Their results were obtained using a Vero4DRT system but assessed from a fixed imaging viewpoint. Although their research focused solely on predicting tumor position errors, our study encompasses both the prediction and evaluation of tumor positions. Recent studies have explored AI‐based tumor or surrogate detection.^14^, ^15^, ^30^, ^31^ Hirai et al. and Lin et al. reported AI‐based methods,^30^, ^31^ with tracking errors of 1.30 ± 0.54 mm^30^ and 2.3 mm (95th percentile in the AP direction), respectively.^31^ However, real‐time AI‐based tracking during beam delivery remains limited by safety concerns, clinical reliability, and regulatory hurdles. Current guidelines from the European Society for Therapeutic Radiology and Oncology and the American Association of Physicists in Medicine do not yet cover AI‐driven automatic beam delivery,^17^ suggesting that the practical implementation of AI in ML‐RTTT radiotherapy will require further time and development.

**TABLE 5:** Comparison of diaphragm‐based ML‐RTTT methods reported in previous studies.

Author	No. of patients	Disease site	AI usage	View	Evaluation times [s]	Evaluation metrics	Errors [mm]
Rostamzadeh *et al*.^12^	10	Liver	No	Fixed	20–40	van Herk^28^, ^29^	(LR, SI, AP) = (2.2, 5.0, 4.7)
Hirai *et al*. ^30^	7	Liver	Yes	Fixed	13	3D mean ± SD	1.30 ± 0.54
Lin *et al*.^31^	10	Lung	Yes	Fixed	25	AP views (2D)	Mean: 1.1 95^th^ percentile: 2.3
This study	23	Lung, liver	No	Rotated	50	3D mean ± SD	3.5 ± 3.4
						90th percentile	(LR, SI, AP) = (2.3, 4.5, 3.7)

Abbreviations: 2D, two‐dimension; 3D, three‐dimension; AI, artificial intelligence; AP, anterior–posterior; LR, left–right; SI, and superior–inferior.

Our method is not limited to the diaphragm but can be applied to other internal structures that show a strong correlation with tumor motion and possess high contrast. The detection templates are created from pCT scans, and the epipolar lines, aiding in the detection of these structures, are derived from the system's geometric information, making them independent of the detected tumor. A study by Spoelstra et al. highlighted the importance of selecting tumor surrogates, emphasizing factors like tumor position, baseline motion, and respiratory phase.^19^ Furthermore, the present approach utilizes a gantry‐mounted, orthogonal dual kV x‐ray imaging system, which facilitates effective lateral imaging, particularly important in lateral chest projections where the shadows of the left and right diaphragms can overlap. This system enables continuous acquisition of kV images synchronized with gantry rotation, allowing real‐time monitoring throughout VMAT delivery. Although such dual kV rotating systems are currently available on a limited number of platforms, the proposed method could be extended to alternative configurations—such as dual kV fixed‐angle systems or single kV rotating systems—assuming accurate diaphragm detection can be achieved. In 2023, Hitachi High‐Tech introduced the OXRAY system,^32^, ^33^ a successor to the Vero4DRT, equipped with RTTT‐VMAT functionality. The diaphragm‐based tracking framework developed in this study has been implemented within the OXRAY platform, where further clinical and technical refinements are actively underway to enable markerless RTTT in conjunction with VMAT.

This study had three main limitations. First, the motion model relies on the rate of the external surrogate breathing signal, which is measured using markers placed on the skin. Because the breathing‐induced motion may be small and superimposed with cardiac pulsations, the signal can be noisy. Taking the derivative of this signal amplifies the noise, which may affect the characterization of the function *F*(*p*) and, consequently, the robustness of the 4D model. While preprocessing steps such as smoothing or filtering are applied to mitigate these effects, residual noise may still contribute to occasional prediction errors. Second, the evaluation period was relatively short, lasting only 50 s. At our institution, the beam delivery time for RTTT‐VMAT for lung tumors (prescribed dose: 50 Gy in four fractions to 95% of the planning target volume) is approximately 130 s, highlighting the need for longer imaging durations to simulate clinical scenarios better. Prolonged treatment times may lead to baseline drift due to factors such as muscle relaxation,^34^, ^35^ potentially compromising the accuracy of the 4DM.^36^ Although our results showed that the positional relationships established during the initial 20 s remained relatively stable throughout the 50 s observation period, the system's behavior beyond this duration remains uncertain. If substantial prediction errors occur, reconstruction of the 4DM or recalibration of the offset vector function may be required.^5^, ^36^ Furthermore, improving the intrinsic accuracy of the 4DM used for tumor position prediction remains an important goal. Third, the kV x‐ray images in this study were acquired exclusively during coplanar imaging sessions. However, in clinical settings involving non‐coplanar beam delivery, the diaphragm is viewed from oblique angles, potentially altering its visualization, especially near the diaphragm apex. When the diaphragm is used as a surrogate, such variability can introduce modeling errors due to inaccuracies in identifying the anatomical location of the diaphragm apex. Given the prevalence of non‐coplanar beam delivery in clinical practice, it is essential to develop methods that can effectively address these imaging challenges.

## 5 CONCLUSION

In this study, we developed an advanced tumor position estimation algorithm for ML‐RTTT radiotherapy that effectively accounts for tumor–surrogate asynchrony using a gantry‐mounted orthogonal kV x‐ray imaging system. By integrating the estimated tumor positions into the 4DM, we implemented and comprehensively evaluated ML‐RTTT‐VMAT. The evaluation demonstrated that the prediction errors of tumor position in the LR, SI, and AP directions were comparable to those obtained without applying an offset vector. This algorithm not only improves the precision of ML‐RTTT radiotherapy but also offers a practical solution for implementing hybrid ML‐RTTT‐VMAT, marking a significant advancement in the field.

## CONFLICT OF INTEREST STATEMENT

Takashi Mizowaki and Mitsuhiro Nakamura received research grants and a scholarship donation from Hitachi High‐Tech Ltd. The other co‐authors participated in collaborative research with Hitachi High‐Tech Corp.

## Data Availability

The data are not publicly available owing to privacy or ethical restrictions.
